# Case Report: dynamic monocyte reprogramming during ALSS therapy in type B HBV-ACLF revealed by single-cell transcriptomics

**DOI:** 10.3389/fimmu.2026.1801893

**Published:** 2026-05-26

**Authors:** Jiale Xie, Jiahua Liu, Feiyu Wang, Dongxuan Zhang, Yuyao Tian, Júlio Ken Matsubara, Jingxiang Zhang, Jie Yin, Zian Chen, Huimin Liu, Xiaoshuang Xu, Xin Wang, Guifen Liu, Jianhua Wang, Yansheng Jia, Lili Chang, Yu Guo, Wei Qi, Yan Wang

**Affiliations:** 1 Department of Gastroenterology, The Second Hospital of Hebei Medical University, Hebei Key Laboratory of Gastroenterology, Hebei Institute of Gastroenterology, Hebei Clinical Research Center for Digestive Diseases, Shijiazhuang, Hebei, China; 2 Hebei Medical University, Shijiazhuang, China; 3 Department of Gastroenterology, Beijing Changping Hospital of Traditional Chinese Medicine, The Affiliated Changping Hospital of Beijing University of Chinese Medicine, Beijing, China; 4 Department of Biomedical Engineering, The Chinese University of Hong Kong, Hong Kong, Hong Kong SAR, China; 5 Marília Medical School, São Paulo, Brazil; 6 Affiliated Hospital of Hebei University of Engineering, Handan, China; 7 The Fifth Hospital of Shijiazhuang City, Shijiazhuang, China; 8 Department of Gastroenterology, Shijiazhuang People’s Hospital, Shijiazhuang, China

**Keywords:** artificial liver support system, case report, HBV-related acute-on-chronic liver failure, monocyte, single-cell RNA sequencing

## Abstract

**Background:**

Hepatitis B virus-related acute-on-chronic liver failure (HBV-ACLF) is characterized by intense systemic inflammation and immune dysregulation, with monocytes playing a central role in its progression. Patients with HBV-ACLF typically receive comprehensive treatment including artificial liver support system (ALSS). Here, we report a case of HBV-ACLF in which single-cell RNA sequencing (scRNA-seq) showing dynamic reprogramming of monocyte upon ALSS treatment.

**Methods:**

We conducted the single-cell and bulk RNA sequencing with peripheral blood mononuclear cells (PBMCs) from a patient with type B HBV-ACLF, both before and after ALSS therapy. The scRNA-seq data were subjected to bioinformatic analyses, including graph-based clustering, pseudotime trajectory inference, and M1/M2 polarization scoring. Findings were further validated using matched bulk transcriptomes.

**Results:**

Among PBMCs, monocytes displayed the most dramatic transcriptomic changes. A distinct inflammatory monocyte subpopulation (Mono4) was identified, featuring upregulated mitochondrial genes, and enrichment of secreted factors associated with platelet activation and systemic inflammation. During the ALSS-based comprehensive treatment course, we observed a decreased proportion of Mono4 and attenuation of its pro-inflammatory secretory signature. Pseudotime analysis further revealed a differentiation trajectory from classical monocytes (Mono1 and Mono2) to Mono4, which decreased with treatment. Consistent with these findings, bulk RNA-seq deconvolution confirmed the monocyte-centric immune landscape.

**Conclusion:**

In this case report, comprehensive treatment including ALSS was linked to monocyte reprogramming and the decreased pro-inflammatory Mono4 subpopulation. Furthermore, Mono4 may play a central role in the systemic hyperinflammatory response.

## Introduction

Hepatitis B virus-related acute-on-chronic liver failure (HBV-ACLF) is a severe clinical syndrome characterized by rapid deterioration of liver function and high mortality, driven by systemic inflammation and immune dysregulation (1, 2). Monocytes, as key mediators of the inflammatory cascade, play a critical role in the pathogenesis of HBV-ACLF, causing both liver inflammation and regeneration (3, 4).

The management of HBV-ACLF poses a significant clinical challenge, as standard medical treatment (SMT) alone is frequently associated with poor outcomes. Currently, liver transplantation is the only method capable of curing severe or advanced HBV-ACLF (5), but its clinical application is limited by a severe shortage of donor organs, high costs, and the narrow therapeutic time window. Artificial liver support system (ALSS) therapy mitigates inflammatory cascade, supports hepatic regeneration, reduces short-term mortality (6, 7), and could serve as a critical bridge to liver transplantation (8). Although previous scRNA-seq studies have characterized monocyte heterogeneity in ACLF progression (3, 9), the therapeutic reprogramming of these subsets by ALSS remains undefined.

To address this gap, we present a case of a young male with Type B HBV-ACLF treated with SMT and ALSS, characterized through scRNA-seq and bulk RNA-seq analyses of PBMCs.

## Methods and materials

### Patient recruitment and diagnosis

The study protocol was approved by the Research Ethics Committee of the Second Hospital of Hebei Medical University (2022-R069). Written informed consent was obtained from the patient prior to sample collection. A patient meeting COSSH-ACLF criteria (TB ≥ 12 mg/dL and INR ≥ 1.5, regardless of cirrhosis) (10) was enrolled, and PBMCs were isolated for bulk and scRNA-seq analysis.

### PBMCs collection

To capture dynamic changes during treatment, peripheral blood was collected before and after each ALSS session. PBMCs were isolated by density gradient centrifugation, washed, counted, and cryopreserved in medium containing 10% DMSO at -80 °C.

**Figure 1:**
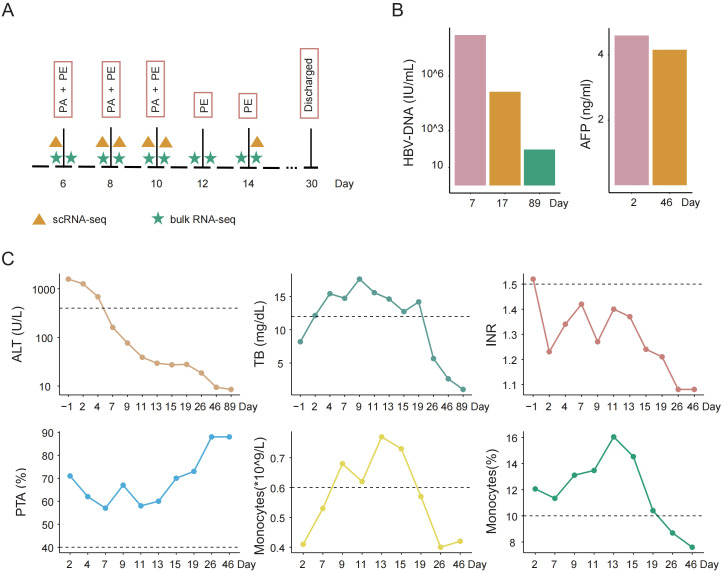
Schematic of clinical treatment and dynamic changes in laboratory parameters. **(A)** timeline showing the patient’s treatment schedule from admission to discharge, as well as sampling points for scRNA-seq and bulk RNA-seq. **(B)** dynamic changes in HBV-DNA and alpha-fetoprotein (AFP) levels. **(C)** dynamic profiles in key laboratory indicators: alanine aminotransferase (ALT), total bilirubin (TB), international normalized ratio (INR), prothrombin time activity (PTA), monocyte percentage, and monocyte count. Dashed lines denote the diagnostic thresholds for HBV-ACLF (liver function and coagulation parameters) and the upper normal limits for complete blood count (CBC).

All ten samples collected throughout the treatment were subjected to bulk RNA-seq, and six were subjected to scRNA-seq (**Figure 1A**; **Supplementary Table 1**).

### Bulk RNA-seq

Total RNA was isolated from PBMCs using TRIzol reagent. Libraries were prepared with the NEB Next Ultra RNA Library Prep Kit (CapitalBio Technology, Beijing, China) and sequenced on an Illumina NovaSeq platform. Reads were aligned to the human GRCh38 genome using HISAT2 v2.2.1. Differentially expressed genes (DEGs) were identified using DESeq2 v1.44.0 in R.

### scRNA-seq library preparation and sequencing

Thawed PBMCs were assessed for viability (>90%), clumping (<5%), and nucleated cell content (>80%). Approximately 10,000 cells per sample were loaded into a microfluidic chip from the Chip A Single Cell Kit v2.0 (MobiDrop, cat. no. S050100201) using the MobiNova-100 system. Single cells and gel beads bearing cell-specific barcoded oligos were co-encapsulated in droplets. After light-induced release of oligos, mRNA capture, reverse transcription, and cDNA amplification, libraries were constructed using the High Throughput Single Cell 3′ RNA-Seq Kit v2.0 and sequenced on an Illumina NovaSeq 6000.

### scRNA-seq data processing

Raw data were processed with Mobivision v3.0 against the GRCh38 reference. Downstream analysis used Seurat v5.3.0 (11) in R v4.4.0. Low-quality cells were filtered (<400 or >6000 genes, <500 UMIs, >10% mitochondrial genes) (**Supplementary Figure 1** and **S2**; **Supplementary Table 2**). Data were normalized (LogNormalize), the top 2000 highly variable genes selected, and scaled. Batches were integrated using Harmony v1.2.1 (12). Clustering was performed on the first 20 principal components at resolution 0.5, followed by uniform manifold approximation and projection (UMAP) visualization. Doublets were removed with DoubletFinder v2.0.4 (13), and ambient RNA contamination was mitigated using DecontX v 1.6.0 (14) (**Supplementary Figure 3**). Following quality control, cells were reclustered and annotated into eight major clusters using FindAllMarkers and cross-referenced with the CellMarker database v2.0 (15) (**Supplementary Figure 4**). Visualizations were generated with ggplot2 v3.5.2.

### DEG identification and functional annotation

DEGs in monocytes post- versus pre-ALSS were identified using FindMarkers (Wilcoxon rank-sum test), with an absolute log_2_ fold change greater than 0.5 and a Benjamini–Hochberg-corrected P value < 0.05. Gene ontology and pathway enrichment were performed with Metascape (www.metascape.org) (16).

### M1/M2 polarization scoring

M1 and M2 scores in monocytes were computed using Seurat’s AddModuleScore. The M1 score incorporated *CD68*, *CD80*, *CD86*, *FCGR1A*, *CLEC7A*, *FCGR2A*, and *FCGR3A*; the M2 score used *CSF1R*, *CD163*, *MRC1*, and *CD209*.

### Trajectory inference and pseudotime analysis

Trajectory inference was performed using Monocle3 v1.4.26 (17). The principal graph was constructed using learn_graph with default parameters and pseudotime calculated via order_cells. The root state was manually set in the Mono1 based on classical monocyte marker expression. Trajectory-associated genes were identified using graph_test (Moran’s I test, q < 0.05).

### Deconvolution of bulk RNA-seq

Immune cell proportions were estimated using CIBERSORT with the LM22 signature matrix, which was derived from purified peripheral blood leukocyte subsets and flow cytometry-validated on PBMC bulk RNA-seq (18).

### SCIPAC integration

Subpopulation-specific treatment associations were identified by integrating bulk and scRNA-seq data with SCIPAC v0.1.0 (19) using default parameters, quantifying links between single-cell profiles and the post-treatment bulk phenotype.

### MacSpectrum online analysis

Monocyte polarization (Macrophage Polarization Index, MPI) and maturation (Activation-Induced Macrophage Differentiation Index, AMDI) were quantified using MacSpectrum (20), which calculates indices based on principal component analysis (PCA) of single-cell transcriptomes relative to reference states, thereby capturing continuous polarization states beyond the binary M1/M2 classification based on predefined marker gene modules. ALSS effects were evaluated by comparing index distributions pre- and post-treatment (https://macspectrum.uconn.edu/).

### Statistics

Statistical methods and thresholds are specified in the text, figures, or legends.

### Case report

Here, we report a case of HBV-ACLF in a 33-year-old male patient with type 2 diabetes mellitus and no significant family history of liver disease. He had been a chronic hepatitis B surface antigen (HBsAg) carrier for more than ten years but with no subsequent management. The patient was hospitalized with acute onset of abdominal distension and marked jaundice.

Prior to admission, laboratory tests from a local hospital revealed severely impaired liver function: alanine aminotransferase (ALT) 1585.89 U/L, aspartate aminotransferase (AST) 959.24 U/L, total bilirubin (TB) 140.22 μmol/L, direct bilirubin (DB) 128.25 μmol/L, and an international normalized ratio (INR) of 1.52. Upon admission, physical examination revealed generalized jaundice of the skin and mucous membranes. Serological tests were positive for hepatitis B surface antigen (HBsAg), hepatitis B e antigen (HBeAg), hepatitis B e antibody (HBeAb), and hepatitis B core antibody (HBcAb). The hepatitis B virus DNA viral load was more than 1.7 × 10^8^ IU/mL. FibroScan showed liver stiffness of 30.9 kPa, and contrast-enhanced CT revealed splenomegaly and no ascites, supporting diagnoses of cirrhosis and portal hypertension. Based on the above clinical presentation, laboratory and imaging findings, he was diagnosed as type B HBV-ACLF, with MELD score of 15.3 and COSSH-ACLF II score of 4.66 (10, 21, 22).

The patient was immediately started on entecavir (1 mg/day) to suppress viral replication, alongside standard supportive care. Although transaminases began to trend downward (ALT to 684.2 U/L; AST to 259.4 U/L), his condition remained critical as TB rose to 263.8 μmol/L and prothrombin time activity (PTA) declined to 62% (**Figure 1C**). Given a diagnosis of COSSH-ACLF Grade 1 with presentation within the optimal window, INR ≥ 1.5, and TB ≥ 10 × ULN, ALSS was initiated on day 6 as extracorporeal liver support (23).

ALSS comprised five sessions: the first three used combined plasma adsorption plus plasma exchange (PA+PE), followed by two sessions of plasma exchange (PE) alone (**Figure 1A**). Sessions were performed every other day based on clinical tolerance and laboratory improvement. The transition from PA+PE to PE was prompted by initial improvements in bilirubin and coagulation parameters after the first three sessions. All procedures were well tolerated. Following five ALSS cycles, a pronounced reduction in HBV-DNA load (from >1.7×10^8^ to 9.32×10¹ IU/mL) was observed, indicating effective virological control, alongside a gradual decrease in α-fetoprotein (AFP) levels (**Figure 1B**). Moreover, monocytes, critical mediators of liver inflammation and regeneration, demonstrated dynamic decrease throughout the disease course after treatment (**Figure 1C**). Clinically, the patient reported significant relief from abdominal distension and jaundice.

**Figure 2:**
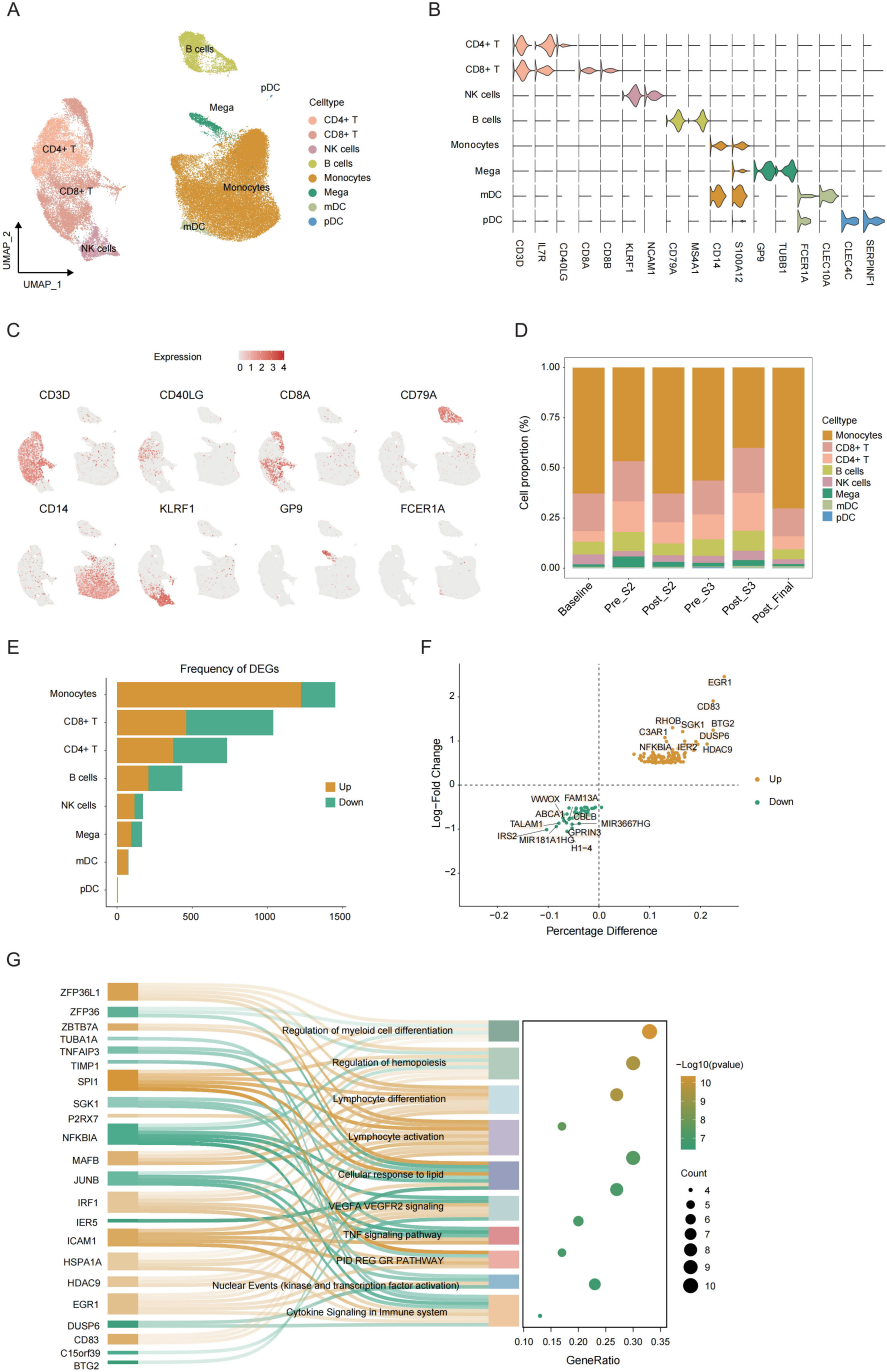
Single-cell analyses of PBMCs in HBV-ACLF and transcriptomic changes of monocytes following ALSS treatment. **(A)** two-dimensional uniform manifold approximation and projection (UMAP) visualization of scRNA-seq data using the patient’s PBMCs. **(B)** violin plot of marker genes for distinct cell types. **(C)** UMAP plot showing cell clusters identified using canonical cell markers. Cell colors represent marker expression levels on a logarithmic scale. **(D)** bar plot shows cell compositions at a single sample level. **(E)** the bar chart shows upregulated and downregulated DEGs in each cell subtype post-treatment versus pre-treatment, identified by |log-fold change| > 0 and adjusted P value < 0.05. **(F)** the difference values and log-fold change of monocytes DEGs. Genes highlighted in yellow and green have |log-fold change| > 0.5 and adjusted P values < 0.05. **(G)** functional enrichment analysis of upregulated DEGs in monocytes. Enriched pathways and biological processes are shown. The color scale and dot size correspond to the -log10 (P value) and gene count, respectively.

To investigate how ALSS therapy modulates the immune response in HBV-ACLF, particularly the role of monocytes, we collected PBMCs and analyzed them using scRNA-seq and bulk RNA-seq (**Figure 1A**). After stringent quality control, UMAP clustering of 55,087 PBMCs identified 17 distinct clusters (**Supplementary Figure 4A**). These populations, annotated based on gene marker expression, included CD4+ and CD8+ T cells, B cells, natural killer (NK) cells, monocytes, megakaryocytes, myeloid dendritic cells (mDCs), and plasmacytoid dendritic cells (pDCs) (**Figures 2A–C**). Monocytes were the predominant subset, comprising 58.2% of PBMCs (**Figure 2D**; **Supplementary Figure 4B**).

Among PBMCs, monocytes exhibited the largest number of DEGs in both comparisons (**Figure 2E**; **Supplementary Figure 5A**). In the peri-session comparison (Post_S2/S3 vs Pre_S2/S3), samples collected immediately before and after ALSS sessions (S2 and S3) captured transcriptional changes temporally aligned with the ALSS window. In contrast, the course-level comparison (Post_Final vs Baseline) reflects cumulative therapeutic impact, including ALSS, antiviral therapy, and supportive care.

Monocyte DEGs showed upregulation of transcripts linked to anti-inflammatory polarization and differentiation. Notably, *SGK1* (associated with M2 polarization) (24) and *DUSP6* (differentiation regulator) (25) were consistently upregulated across both comparisons (**Figure 2F**; **Supplementary Figure 5B**). Metascape enrichment highlighted shared biological processes, including “Regulation of myeloid cell differentiation” and “Regulation of hemopoiesis” (**Figure 2G**; **Supplementary Figure 5C**), consistent with immune reconstitution.

However, distinct patterns emerged between analyses. In the peri-session analysis, monocytes showed increased expression of early anti-inflammatory transcription factor *EGR1* (26) and inflammation-resolution checkpoint *CD83* (27) (**Figure 2F**), with enriched pathways including “TNF signaling pathway” and “Lymphocyte differentiation” (**Figure 2G**). Collectively, these data reveal rapid anti-inflammatory transcriptional reprogramming in monocytes immediately following ALSS sessions. While promising, the single-patient design precludes definitive causal attribution, as concomitant treatments may also play a role.

Conversely, the course-level comparison showed sustained upregulation of anti-inflammatory factor *PPARG* (28) and negative regulators of inflammation *ZFP36* and *ZFP36L1* (29) (**Supplementary Figure 5B**), alongside enrichment of “Positive regulation of mRNA catabolic process” and “Positive regulation of fat cell differentiation” (**Supplementary Figure 5C**), suggesting gradual shifts toward immunometabolic and post-transcriptional regulatory programs.

**Figure 3:**
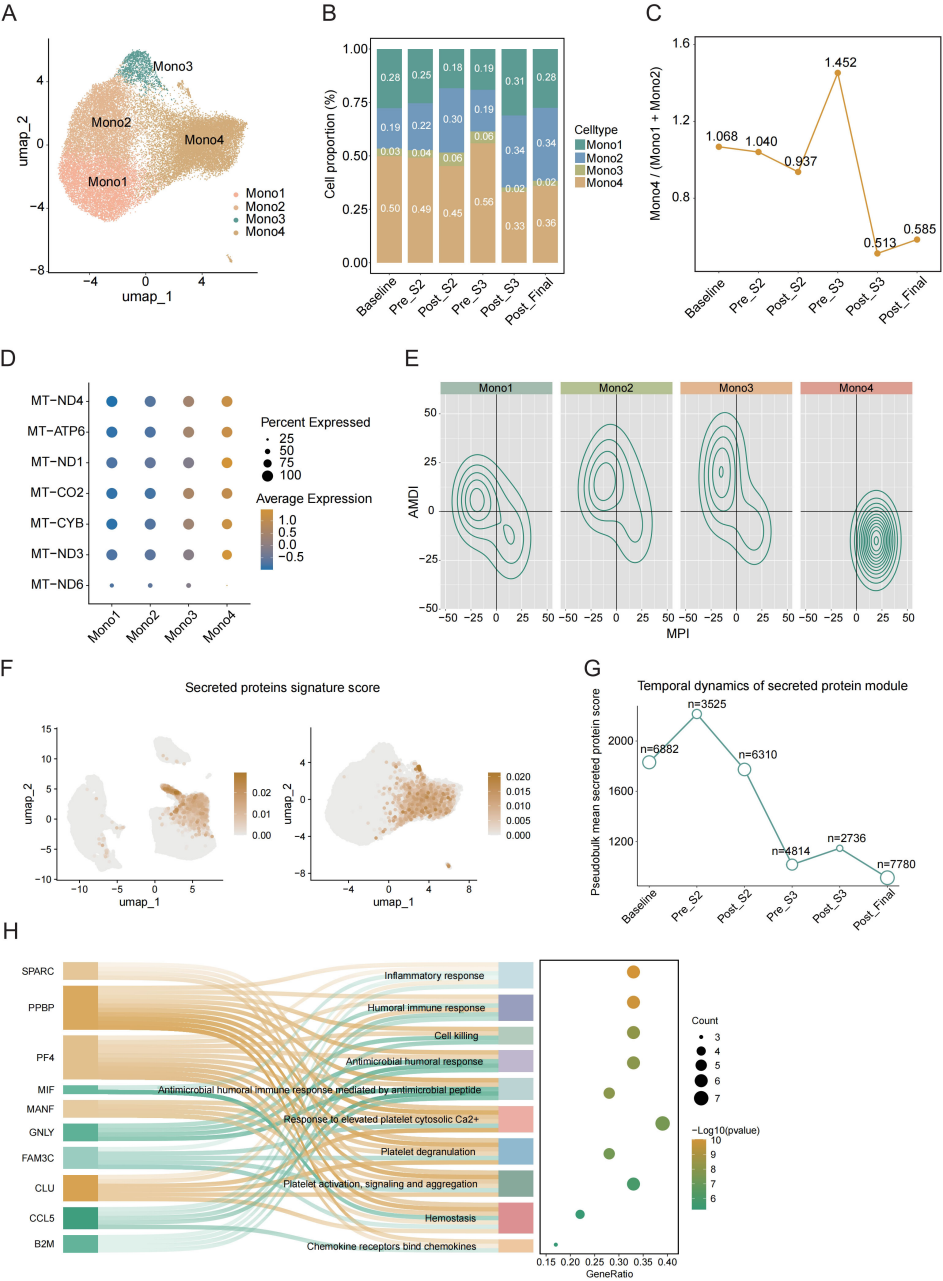
Single-cell profiling of monocyte heterogeneity, dynamics, and function during ALSS treatment in HBV-ACLF. **(A)** UMAP visualizations depict the spatial clustering and distribution of monocyte subpopulations, with each dot representing a cell and different colors representing different subpopulations. **(B)** the bar charts display the proportion of monocyte subpopulations across different sampling time points. **(C)** dynamics of the monocyte subset ratio (Mono4/(Mono1+Mono2)) upon ALSS therapy. **(D)** the average expression of mitochondrial genes in each monocyte subpopulation. The size of dot represents the number of mitochondrial genes, and the color intensity represents the average expression levels of corresponding genes. **(E)** radar charts for four monocyte subpopulations across all sampling time points, displaying the macrophage polarization index (MPI) and activation-induced macrophage differentiation index (AMDI). **(F)** UMAP projection colored by secreted protein signature scores (UniProt gene set). **(G)** temporal dynamics of the secreted gene module in pseudobulk data, shown as the mean module score per time point. Point size is proportional to the number of cells captured at each timepoint (n values labeled above each point). No formal statistical testing was performed due to lack of biological replicates. **(H)** functional enrichment analysis of secreted proteins upregulated in Mono4 (log-fold change > 0.25 and an adjusted P value < 0.05). Significantly enriched pathways and biological processes are shown. The color scale and dot size correspond to the -log10 (P value) and gene count, respectively. The x-axis (gene ratio) represents the fraction of genes associated with each term.

To further clarify the monocytes reprogramming, monocytes were reclustered into four subpopulations (**Figure 3A**; **Supplementary Figure 6A**). Mono4 displayed high levels of *CCL5*, *PF4*, and *PPBP*, whereas *ITGA2B*, *VWF*, *GP9*, and *GATA1* were barely detected (**Supplementary Figure 6B**). *CCL5* expression progressively declined during ALSS therapy (**Supplementary Figure 6C**). It was notably found that a decrease in Mono4 subpopulation after ALSS treatment (**Figure 3B**). This trend was quantified by the Mono4/(Mono1+Mono2) ratio, which decreased from 1.068 to 0.585 (**Figure 3C**), underscoring a selective depletion of the Mono4 subset from the monocyte pool.

Moreover, a potential abnormal metabolic activation was revealed by highly expressed mitochondrial genes in Mono4 subpopulation (e.g., *MT-ND3*, *MT-CYB*), which was consistent with cellular stress response in an inflammatory environment (**Figure 3D**).

Pseudobulk analysis of monocyte M1/M2 ratios showed variable changes during ALSS, with no overall decrease from baseline to the final time point (**Supplementary Figure 6D**). To further delineate monocyte polarization states, MacSpectrum analysis was performed using the Macrophage Polarization Index (MPI) and Activation-induced Macrophage Differentiation Index (AMDI). AMDI values increased gradually from Mono1 to Mono3 with mild MPI changes, while Mono4 was positioned in the fourth quadrant (**Figure 3E**). MPI values in Mono1–Mono3 fluctuated but slightly decreased during treatment (**Supplementary Figure 6E** and **S7**).

To identify potential extracellular communication patterns, secreted protein signatures (based on UniProt gene sets) were calculated. These signatures were predominantly enriched in monocytes (**Figures 3F, left**), with particularly high in the Mono4 subpopulation (**Figures 3F, right**). Temporal dynamics of the secreted protein signature score were evaluated using pseudobulk aggregation across six timepoints, revealing a general decrease during the treatment course (**Figure 3G**). Functional enrichment analysis showed that Mono4-specific secreted proteins were significantly enriched in “Platelet degranulation”, “Platelet activation, signaling and aggregation”, and “Antimicrobial humoral immune response mediated by antimicrobial peptide” pathways (**Figure 3H**).

**Figure 4:**
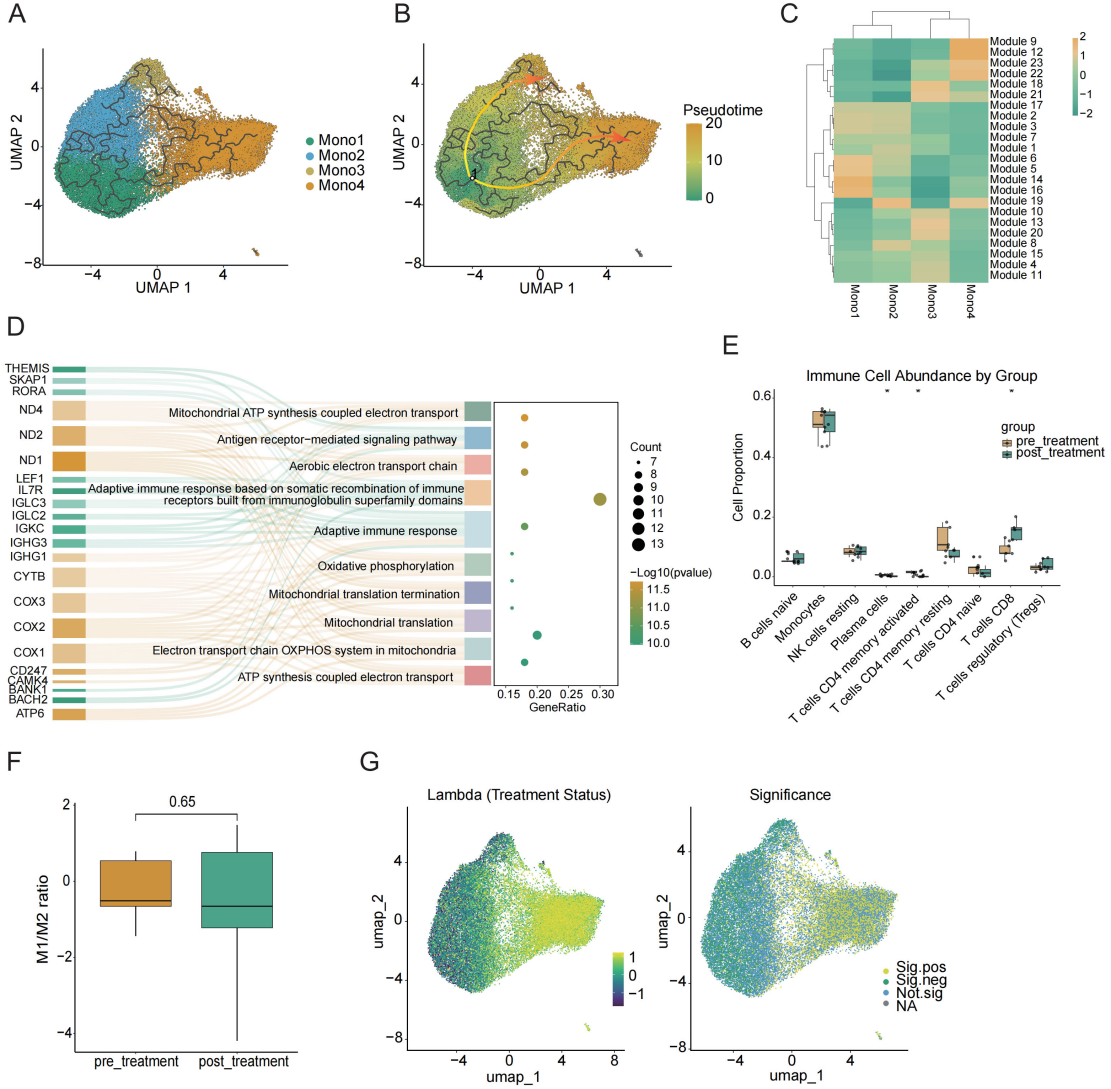
Pseudotime analysis of monocytes subpopulations and bulk transcriptomics of systemic immunity. **(A)** UMAP visualization of monocyte subpopulations (Mono1-Mono4) with the inferred differentiation trajectory. **(B)** cells colored by pseudotime values inferred with Monocle 3. The root was manually selected in the Mono1 according to classical monocyte markers. Arrows indicate the overall trajectory direction from early (Mono1/Mono2) to late pseudotime (Mono4). **(C)** the expression patterns of each gene modules across different monocyte subpopulations. The colors scale represents Z-score normalized gene expression levels, with green denotes low expression and yellow denotes high expression. **(D)** functional enrichment analysis of highly expressed genes in gene modules 9 and 12 within Mono4 subpopulation. Enriched pathways and biological processes are shown. The color scale and dot size correspond to the -Log10 (P value) and gene count, respectively. **(E)** Changes in immune cell proportions inferred by CIBERSORT. (pre-treatment, n = 5; post-treatment, n = 5). Statistical significance was determined using the Wilcoxon rank-sum test (p < 0.05). **(F)** Comparison of M1/M2 ratios before and after treatment using a t-test statistical method. Ratios were calculated from GSVA enrichment scores. **(G)** SCIPAC analysis integrating bulk RNA-seq (post- versus pre-ALSS) with matched single-cell monocyte profiles. (Left) per-cell lambda values, where higher values (yellow) indicate stronger transcriptional similarity to the post-treatment bulk phenotype (i.e., the gene expression pattern that best distinguishes post-ALSS from pre-ALSS samples). (Right) cells colored by statistical significance of the association: yellow (Sig.pos), green (Sig.neg), blue (Not.sig).

Pseudotime trajectory analysis revealed a continuous differentiation path starting from Mono1 (early stage) and progressing to Mono4 (late stage) (**Figures 4A, B**). Gene modules associated with Mono4 (Modules 9 and 12) were significantly enriched in pathways related to mitochondrial energy metabolism and adaptive immune responses, corroborating the high mitochondrial gene expression observed earlier (**Figures 4C, D**).

Bulk RNA-seq deconvolution (CIBERSORT) confirmed the monocyte-heavy landscape and identified significant shifts in the broader immune environment (**Figure 4E**). Following treatment, we observed a decrease in plasma cells and resting CD4+ memory T cells, alongside a notable increase in CD8+ T cells. Monocyte polarization was quantified through GSVA-based M1/M2 enrichment ratios using the same signature genes as in scRNA-seq. Consistent with our scRNA-seq data (**Supplementary Figure 6D**), no significant decrease in the M1/M2 ratio was observed following treatment (**Figure 4F**).

Finally, to characterize the treatment-responsive cell populations, we used SCIPAC (Single-Cell and bulk data-based Identifier for Phenotype Associated Cells) (19) to integrate bulk RNA-seq data from post- versus pre-ALSS PBMCs with our pre-annotated single-cell profiles (**Supplementary Figure 8**). SCIPAC projects a bulk-derived logistic regression model onto the single-cell data and quantifies each cell’s transcriptional association with the post-treatment phenotype. A higher Lambda value indicates stronger similarity between a cell’s gene expression profile and the transcriptional state characteristic of post-ALSS samples. Notably, Mono4 showed the strongest positive association with the post-treatment state, while Mono1-Mono3 were negatively or insignificantly associated (**Figure 4G**).

## Discussion

In this study, we investigated the immunological effects of ALSS therapy in a patient with type B HBV-ACLF, through integrated single-cell and bulk RNA sequencing of PBMCs, with a particular focus on the functional state transitions of monocyte subsets. We observed a reduction in the proportion of an inflammatory monocyte cluster (Mono4) and associated transcriptional shifts during the ALSS-based comprehensive treatment course. These changes coincided with improvements in liver function and clinical outcomes, indicating that ALSS may mitigate systemic inflammation in HBV-ACLF through modulation of monocyte heterogeneity and functional states.

Monocytes were the predominant cell type within PBMCs. They exhibited the most pronounced transcriptomic changes, with the highest number of DEGs. Gene set enrichment analysis revealed upregulation of genes (e.g., *SGK1*, *DUSP6*) associated with anti-inflammatory responses and differentiation, highlighting monocytes as key players in the immunological response to ALSS. Previous reports have established that ALSS eliminates toxins, reduces circulating cytokines such as *IL-6* and *TNF-α*, and improves short-term survival (30, 31). Plasma exchange (PE) has similarly been shown to influence monocyte function in autoimmune diseases (32). Consistent with prior reports of extracorporeal liver support influencing circulating mediators, our data show monocyte transcriptomic changes compatible with functional reprogramming. However, because the patient also received concomitant antiviral therapy and supportive care, these changes should be interpreted as temporal associations rather than definitive effects of ALSS alone.

Several lines of evidence indicate that Mono4 represents a biologically and clinically meaningful proinflammatory monocyte population in HBV-ACLF. Firstly, pseudotime trajectory analysis placed this cluster at the terminal end of monocyte differentiation, its proportion fell from 50% to 36% during ALSS, while the clinical status improved. Secondly, Mono4 showed prominent upregulation of mitochondrial genes (e.g., *MT-ND3*, *MT-CYB*). Given that systemic inflammation in ACLF impairs mitochondrial metabolism and bioenergetics in immune cells (33), this signature probably reflects high cell energy requirements. However, such compensatory mitochondrial activity in a proinflammatory state frequently proves maladaptive: resulting in functional impairment, structural damage, and excess reactive oxygen species production (34). MacSpectrum scoring supported its proinflammatory profile. Mono4 maintained percent.mt below the 10% filtering threshold and exhibited lower nFeature_RNA and nCount_RNA, consistent with a stressed metabolic state. Accordingly, Mono4 appears to undergo mitochondrial distress that could exacerbate systemic inflammation. Additionally, Mono4 was further characterized by a strong secretory phenotype, with enrichment of pathways involving platelet activation and inflammatory responses. Mono4 specifically expressed platelet-derived chemokines *PF4*, *PPBP*, and *CCL5*, but showed low expression of megakaryocyte-specific markers such as *VWF* and *GP1BA*. These features are unlikely to be technical artifacts, given stringent quality control with DecontX for ambient RNA removal and DoubletFinder for doublet exclusion. This signature aligns with prior reports of monocyte-platelet aggregate (MPA)-like subpopulations in chronic HBV-associated fibrosis and ACLF progression (9, 35), reflecting platelet-mediated monocyte reprogramming toward proinflammatory states (36). Mono4 abundance, *CCL5* expression, and secretory protein scores declined progressively during ALSS therapy, indicating reversibility of this activated phenotype. While this reduction correlated with clinical improvement, its value as a predictive biomarker remains exploratory. Finally, SCIPAC analysis revealed that Mono4 exhibited the strongest positive transcriptional association with the post-treatment state. This indicates that, although the proportion of Mono4 decreased following ALSS, its gene expression signature underwent the most pronounced reprogramming and contributed most strongly to the overall shift from the pre-ALSS to the post-ALSS bulk transcriptome. Collectively, these results position Mono4 as a proinflammatory MPA-like population that is highly responsive to effective therapy in HBV-ACLF.

In the context of prior work, our previous type A HBV-ACLF case highlighted a broad shift in monocyte polarization (37), whereas this type B case reveals a more granular, subset-specific response centered on Mono4. This pattern aligns with stage-specific monocyte states delineated by Yao et al. in ACLF (9) and with recent longitudinal single-cell studies identifying inflammatory monocyte modules linked to poor outcomes in HBV-ACLF (3). Similar immunometabolic reprogramming of monocytes has also been observed in HBV-related hepatocellular carcinoma (38), reinforcing the notion that effective therapies in HBV-ACLF may function by selectively targeting and attenuating distinct pathogenic monocyte subsets.

This study provides, to our knowledge, the first single-cell transcriptomic analysis of the effects of ALSS on monocyte heterogeneity in HBV-ACLF, although several limitations should be acknowledged. The single-patient design and concurrent therapies preclude definitive attribution to ALSS alone and restrict clinical generalizability. Although LM22 performs robustly for major PBMC populations compared with other methods (18, 39), its microarray-derived signatures may not fully translate to RNA-seq data, and the inclusion of granulocyte signatures (which are absent in PBMCs) may further limit resolution at the subset level. Of note, pseudotime trajectory inference is highly sensitive to the choice of manual root cell selection and should therefore be interpreted as hypothesis-generating rather than definitive.

Future studies with larger cohorts and strict longitudinal sampling are needed to validate the functional role of Mono4, its response to ALSS, and its potential as a treatment efficacy biomarker. Consistent with precision medicine (40), proteomic validation, mitochondrial/ROS assays, and *in vitro*/ex vivo functional studies will also be essential.

## Conclusion

In conclusion, ALSS-based comprehensive therapy dynamically reprogrammed monocyte heterogeneity, marked by a reduction in the pro-inflammatory Mono4 subset with metabolic stress features. These findings provide mechanistic insights into the immunomodulatory effects of ALSS and highlight the potential for targeted monocyte-based interventions in acute-on-chronic liver failure. However, prospective studies are needed to validate these observations.

## Data Availability

The raw sequence data reported in this paper have been deposited in the Genome Sequence Archive in National Genomics Data Center, China National Center for Bioinformation/Beijing Institute of Genomics, Chinese Academy of Sciences (GSA-Human: HRA016018) that are publicly accessible at https://ngdc.cncb.ac.cn/gsa-human (41, 42).

## Supplementary material

Supplementary Table 1 scRNA-seq sampling time points across ALSS sessions. Abbreviation: ALSS, Artificial Liver Support Systems. Note: Bulk RNA-seq included all 10 samples (all ALSS sessions); scRNA-seq was performed on 6 selected timepoints (Baseline, S2, S3 peri-sessions, and Post_Final).

Supplementary Table 2 Summary of per-sample scRNA-seq quality control metrics. M: million (10^6^ reads); % MT: median percentage of mitochondrial gene expression after basic QC; Est.: estimated; Overall retention rate = Final cells (after DecontX)/Raw cells × 100%.

Supplementary Figure 1 Distribution of quality control metrics across samples after initial filtering. (A) nFeature_RNA, (B) nCount_RNA, and (C) percentage of mitochondrial genes (percent.mt).

Supplementary Figure 2 Distribution of quality control metrics across clusters after initial filtering. (A) nFeature_RNA, (B) nCount_RNA, and (C) percentage of mitochondrial genes (percent.mt).

Supplementary Figure 3 Quality control workflow for doublet and ambient RNA removal. (A-B) UMAP visualization of clusters before and after the complete quality control pipeline. (A) Initial clustering showing widespread ambient RNA contamination (scattered peripheral cells) and unresolved doublet populations. (B) Following sequential doublet detection and ambient RNA correction, the dataset exhibits improved cluster delineation and removal of diffuse peripheral signals. (C-E) Intermediate steps of the doublet detection and removal process. (C) Optimization of the pK parameter using the find.pK() function. (D) Classification of predicted doublets based on the selected pK value. (E) UMAP after doublet exclusion but prior to ambient RNA removal.

Supplementary Figure 4 Cell type annotation and composition of PBMCs. (A) UMAP visualization of 55,087 PBMCs, showing 17 distinct clusters and their annotation into major immune cell types: monocytes (clusters 0,1,2,5,11,15), CD8+ T cells (4,7,8,14), CD4+ T cells (3), B cells (6,12), NK cells (9), megakaryocytes (10), myeloid dendritic cells (mDC, 13), and plasmacytoid dendritic cells (pDC, 16). (B) Donut chart shows the average proportions of each cell subset within PBMCs. (C) Dot plot of marker genes for distinct cell types.

Supplementary Figure 5 Effects of comprehensive treatment on the PBMCs immune profile and monocyte function. (A) The number of DEGs in major immune cell types following comprehensive treatment, identified by |log-fold change| > 0 and an adjusted P value < 0.05 based on the Wilcoxon rank-sum test. (B) ΔPercentage difference and log-fold change based on the Wilcoxon rank-sum test results for differential gene expression comparing pre-treatment and post-treatment in monocytes. Genes highlighted in yellow (upregulated) or green (downregulated) have |log-fold change| > 0.5 and adjusted p < 0.05. (C) Functional enrichment analysis of upregulated DEGs in monocytes after comprehensive treatment (|log-fold change| > 0.5 and adjusted P value < 0.05). The color scale and dot size correspond to the -Log10 (P value) and gene count, respectively. The x-axis (Gene Ratio) represents the fraction of genes associated with each term.

Supplementary Figure 6 Signatures, secretory phenotypes, and polarization dynamics of monocyte subsets during ALSS therapy. (A) Dot plot showing signature genes of monocyte subsets. (B) Dot plot showing secreted chemokines and platelet-associated genes in monocyte subpopulations. (C) Dot plot showing CCL5 expression across treatment time points. (D) Pseudobulk analysis of M1/M2 ratios in monocytes at different time points. Ratio was computed as (mean M1 module score)/(mean M2 module score) after per-timepoint averaging. Point size reflects cell number per timepoint. Dashed line denotes M1/M2 = 1. (E) Radar charts for each monocyte subset before (yellow lines) and after (green lines) comprehensive treatment, showing changes in the Monocyte Polarization Index (MPI) and the Differentiation Index (AMDI). Percentages denote the proportion of cells within each subset pre- (B) and post- (A) treatment.

Supplementary Figure 7 Radar charts displaying the distribution of six time points for each monocyte subset (Mono1 to Mono4).

Supplementary Figure 8 SCIPAC integration of matched bulk and single-cell RNA-seq datasets from PBMCs. (A) per-cell lambda values. (B) Cells colored by significance.
