# Supplementary material for: Case Report: dynamic monocyte reprogramming during ALSS therapy in type B HBV-ACLF revealed by single-cell transcriptomics
Source: Front Immunol. 2026 May 26;17:1801893. doi: 10.3389/fimmu.2026.1801893 (PMC13246407; doi:10.3389/fimmu.2026.1801893)
Supplement: Supplementary Table 1 — scRNA-seq sampling time points across ALSS sessions. Abbreviation: ALSS, Artificial Liver Support Systems. Note: Bulk RNA-seq included all 10 samples (all ALSS sessions); scRNA-seq was performed on 6 selected timepoints (Baseline, S2, S3 peri-sessions, and Post_Final). [file Table1.docx]

**Supplementary Table S1**

scRNA-seq sample collection time points across ALSS sessions.

| **Sample ID** | **ALSS session** | **Collection time point** | **Hospital day** | **Comparison type** |
| --- | --- | --- | --- | --- |
| Baseline | Before S1 | Before 1st ALSS session | Day 6 | course-level |
| Pre_S2 | S2 | Immediately before 2nd ALSS session | Day 8 | peri-session |
| Post_S2 | S2 | Immediately after 2nd ALSS session | Day 8 | peri-session |
| Pre_S3 | S3 | Immediately before 3rd ALSS session | Day 10 | peri-session |
| Post_S3 | S3 | Immediately after 3rd ALSS session | Day 10 | peri-session |
| Post_Final | After S5 | After 5th (final) ALSS session | Day 14 | course-level |

Abbreviation: ALSS, Artificial Liver Support Systems. Note: Bulk RNA-seq included all 10 samples (all ALSS sessions); scRNA-seq was performed on 6 selected timepoints (Baseline, S2, S3 peri-sessions, and Post_Final).

**Supplementary Table S2**

Per-sample standard scRNA-seq quality metrics and filtering summary.

| **Sample ID** | **Total raw reads (M)** | **Mean reads per cell** | **Median genes per cell** | **Median % MT** | **Est. doublet rate (%)** | **Cells (raw)** | **Cells (post-QC)** | **Final cells** | **Overall retention (%)** |
| --- | --- | --- | --- | --- | --- | --- | --- | --- | --- |
| Baseline | 436 | 24,878 | 1,968 | 3.51 | 9.63 | 13,653 | 13,222 | 10,979 | 80.4 |
| Pre_S2 | 433 | 29,104 | 1,593 | 4.12 | 7.44 | 11,173 | 10,992 | 7,559 | 67.7 |
| Post_S2 | 410 | 26,501 | 1,909 | 4.13 | 3.25 | 11,082 | 10,814 | 10,053 | 90.7 |
| Pre_S3 | 434 | 18,505 | 1,081 | 3.75 | 5.57 | 14,630 | 14,371 | 8,559 | 58.5 |
| Post_S3 | 421 | 37,052 | 2,018 | 4.15 | 3.63 | 8,452 | 8,189 | 6,836 | 80.9 |
| Post_Final | 410 | 26,867 | 2,203 | 3.27 | 4.83 | 12,196 | 11,879 | 11,101 | 91.0 |

M: million (10⁶ reads); % MT: median percentage of mitochondrial gene expression after basic QC; Est.: estimated; Overall retention rate = Final cells (after DecontX) / Raw cells × 100%.
