# Supplementary figures and images for: Case Report: dynamic monocyte reprogramming during ALSS therapy in type B HBV-ACLF revealed by single-cell transcriptomics
Source: Front Immunol. 2026 May 26;17:1801893. doi: 10.3389/fimmu.2026.1801893 (PMC13246407; doi:10.3389/fimmu.2026.1801893)

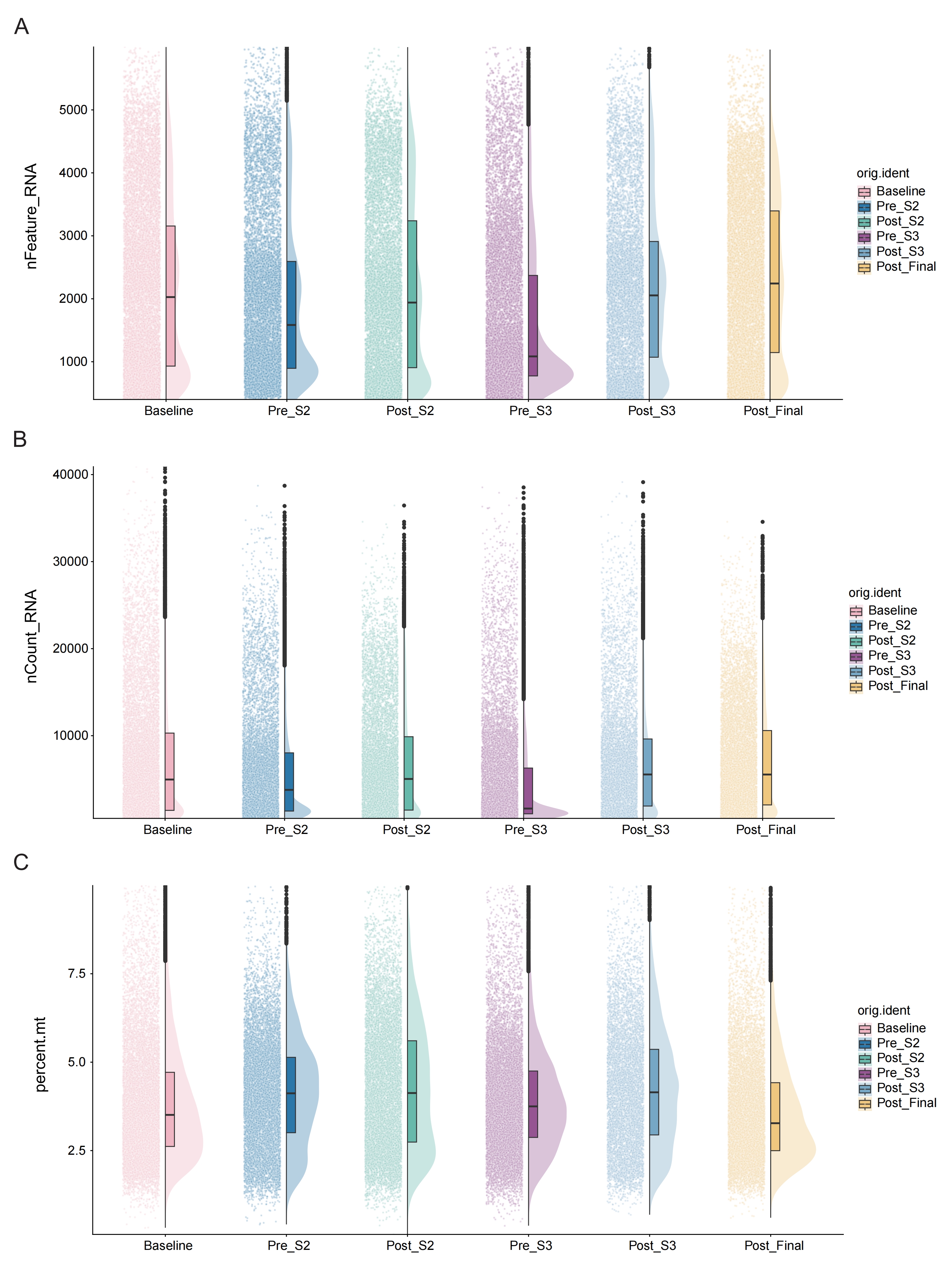

Supplement: Supplementary Figure 1 — Distribution of quality control metrics across samples after initial filtering. (A) nFeature_RNA, (B) nCount_RNA, and (C) percentage of mitochondrial genes (percent.mt). [file Image1.jpeg]

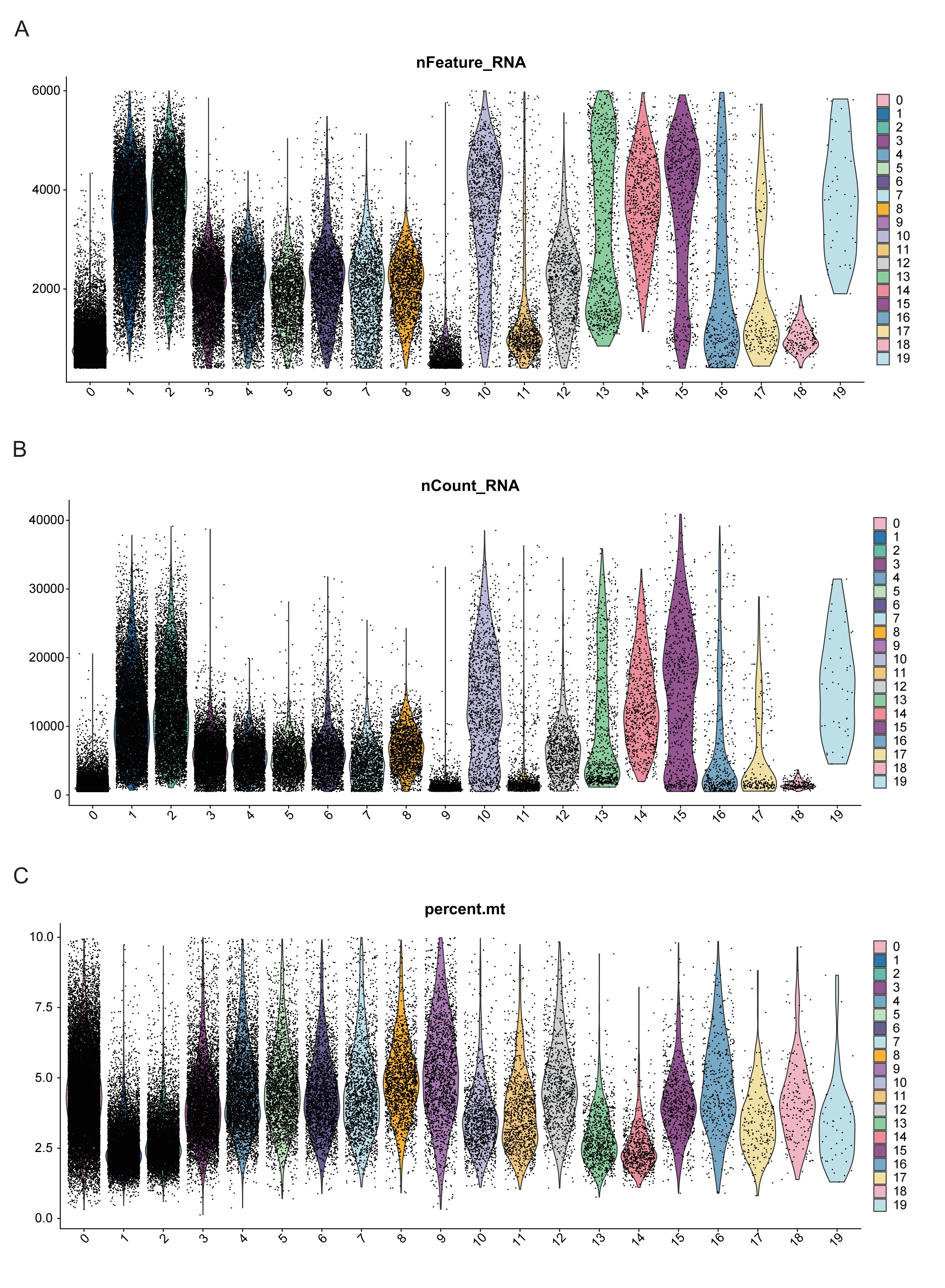

Supplement: Supplementary Figure 2 — Distribution of quality control metrics across clusters after initial filtering. (A) nFeature_RNA, (B) nCount_RNA, and (C) percentage of mitochondrial genes (percent.mt). [file Image2.jpeg]

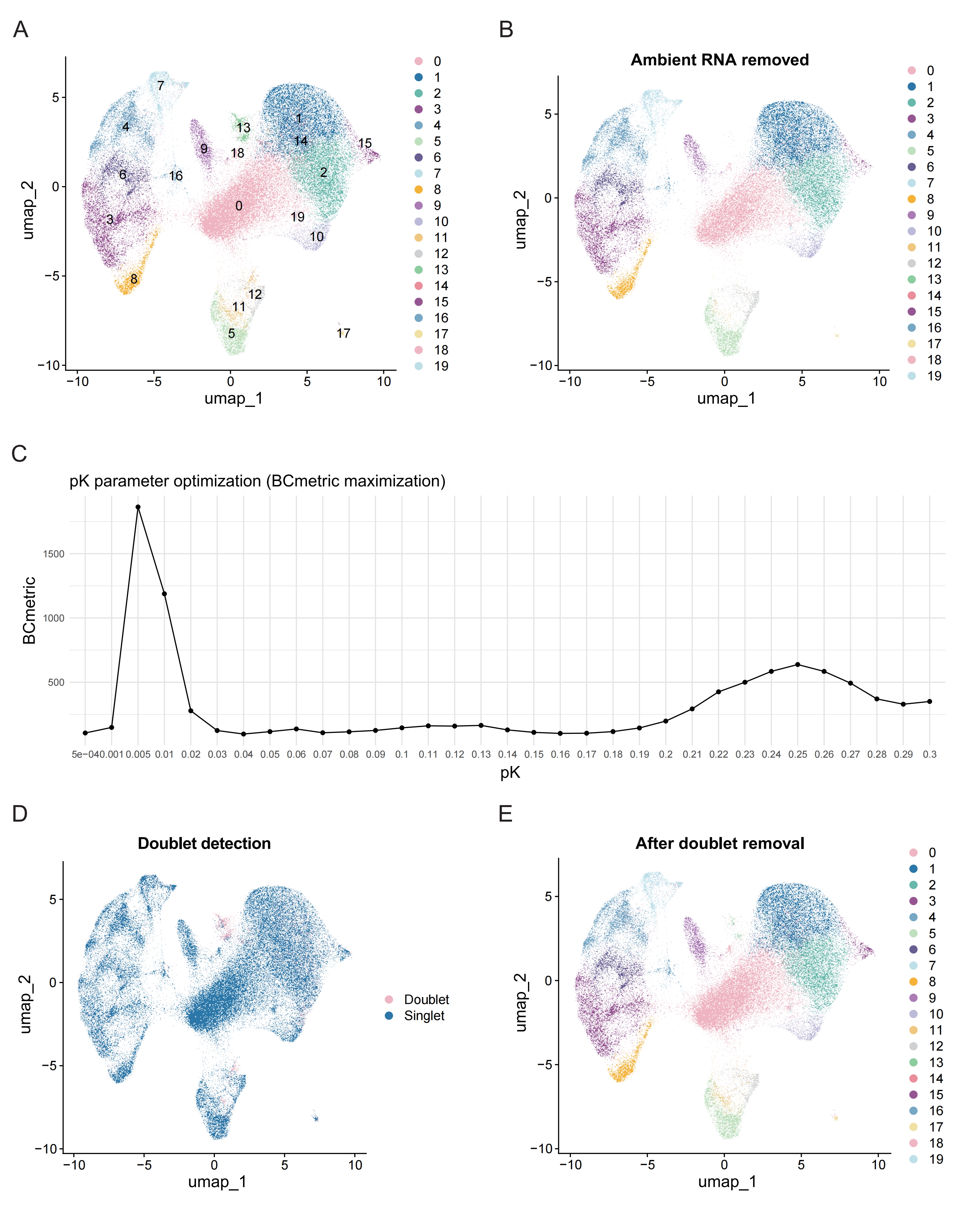

Supplement: Supplementary Figure 3 — Quality control workflow for doublet and ambient RNA removal. (A-B) UMAP visualization of clusters before and after the complete quality control pipeline. (A) Initial clustering showing widespread ambient RNA contamination (scattered peripheral cells) and unresolved doublet populations. (B) Following sequential doublet detection and ambient RNA correction, the dataset exhibits improved cluster delineation and removal of diffuse peripheral signals. (C-E) Intermediate steps of the doublet detection and removal process. (C) Optimization of the pK parameter using the find.pK() function. (D) Classification of predicted doublets based on the selected pK value. (E) UMAP after doublet exclusion but prior to ambient RNA removal. [file Image3.jpeg]

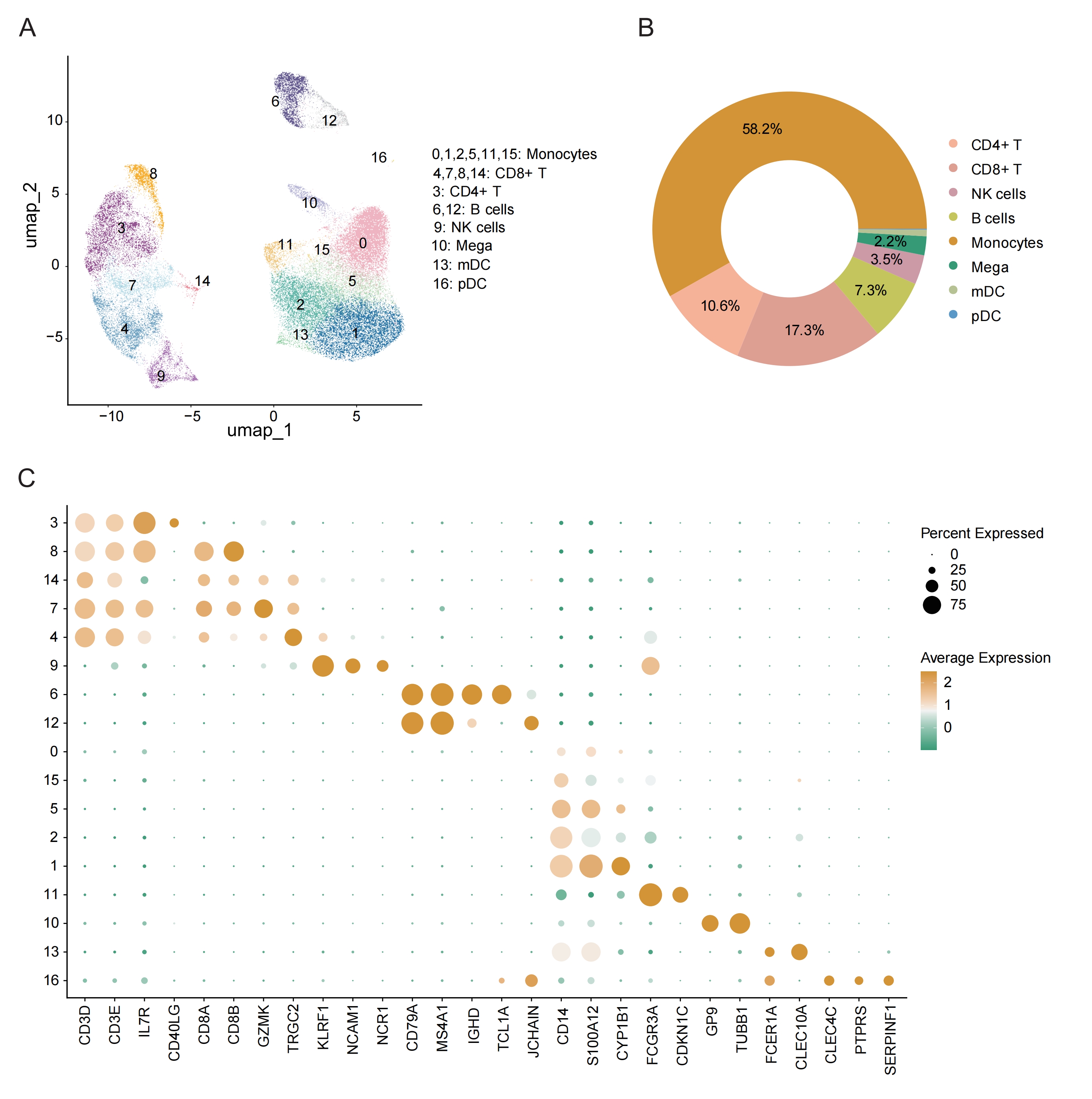

Supplement: Supplementary Figure 4 — Cell type annotation and composition of PBMCs. (A) UMAP visualization of 55,087 PBMCs, showing 17 distinct clusters and their annotation into major immune cell types: monocytes (clusters 0,1,2,5,11,15), CD8+ T cells (4,7,8,14), CD4+ T cells (3), B cells (6,12), NK cells (9), megakaryocytes (10), myeloid dendritic cells (mDC, 13), and plasmacytoid dendritic cells (pDC, 16). (B) Donut chart shows the average proportions of each cell subset within PBMCs. (C) Dot plot of marker genes for distinct cell types. [file Image4.jpeg]

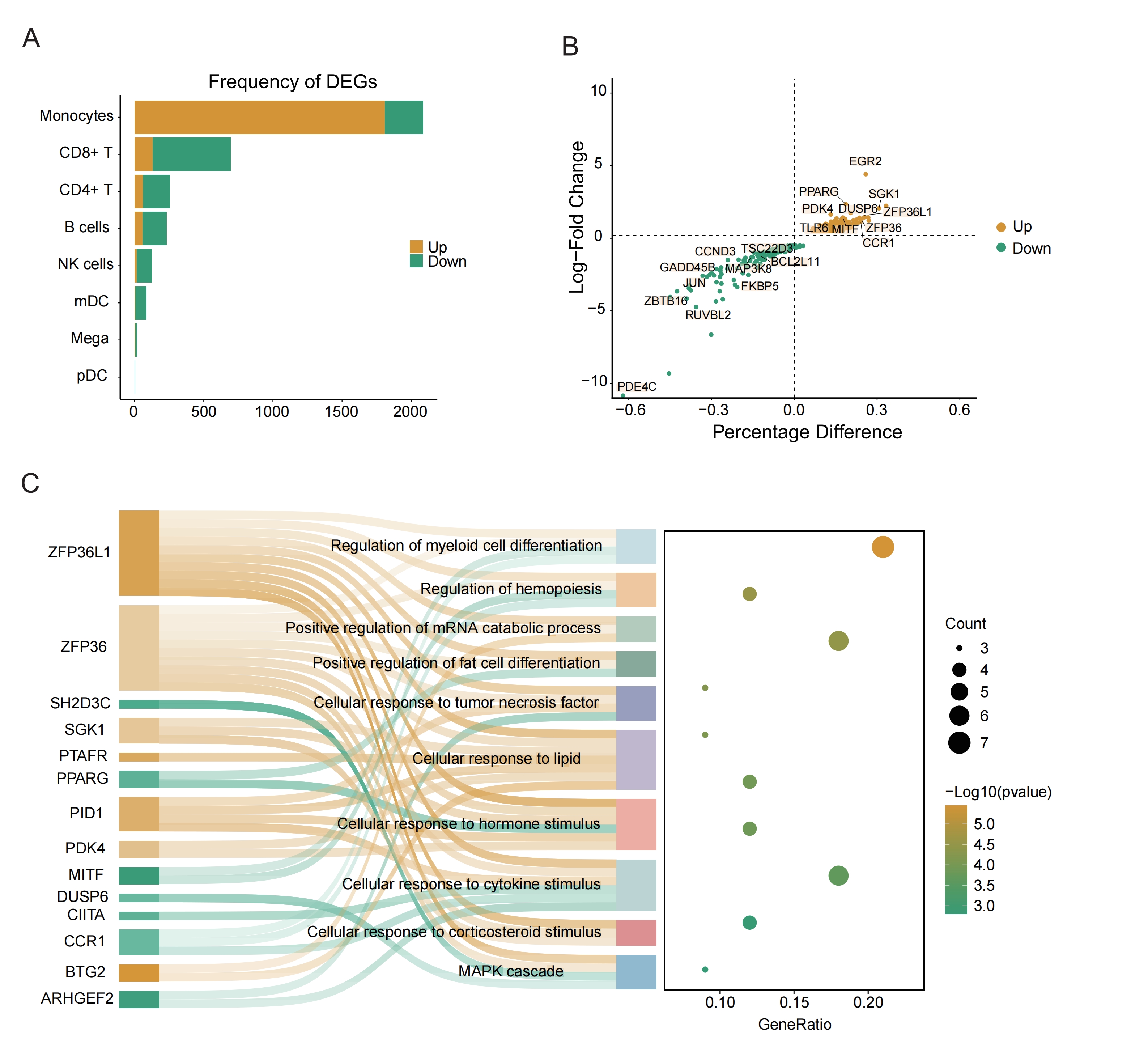

Supplement: Supplementary Figure 5 — Effects of comprehensive treatment on the PBMCs immune profile and monocyte function. (A) The number of DEGs in major immune cell types following comprehensive treatment, identified by |log-fold change| > 0 and an adjusted P value < 0.05 based on the Wilcoxon rank-sum test. (B) ΔPercentage difference and log-fold change based on the Wilcoxon rank-sum test results for differential gene expression comparing pre-treatment and post-treatment in monocytes. Genes highlighted in yellow (upregulated) or green (downregulated) have |log-fold change| > 0.5 and adjusted p < 0.05. (C) Functional enrichment analysis of upregulated DEGs in monocytes after comprehensive treatment (|log-fold change| > 0.5 and adjusted P value < 0.05). The color scale and dot size correspond to the -Log10 (P value) and gene count, respectively. The x-axis (Gene Ratio) represents the fraction of genes associated with each term. [file Image5.jpeg]

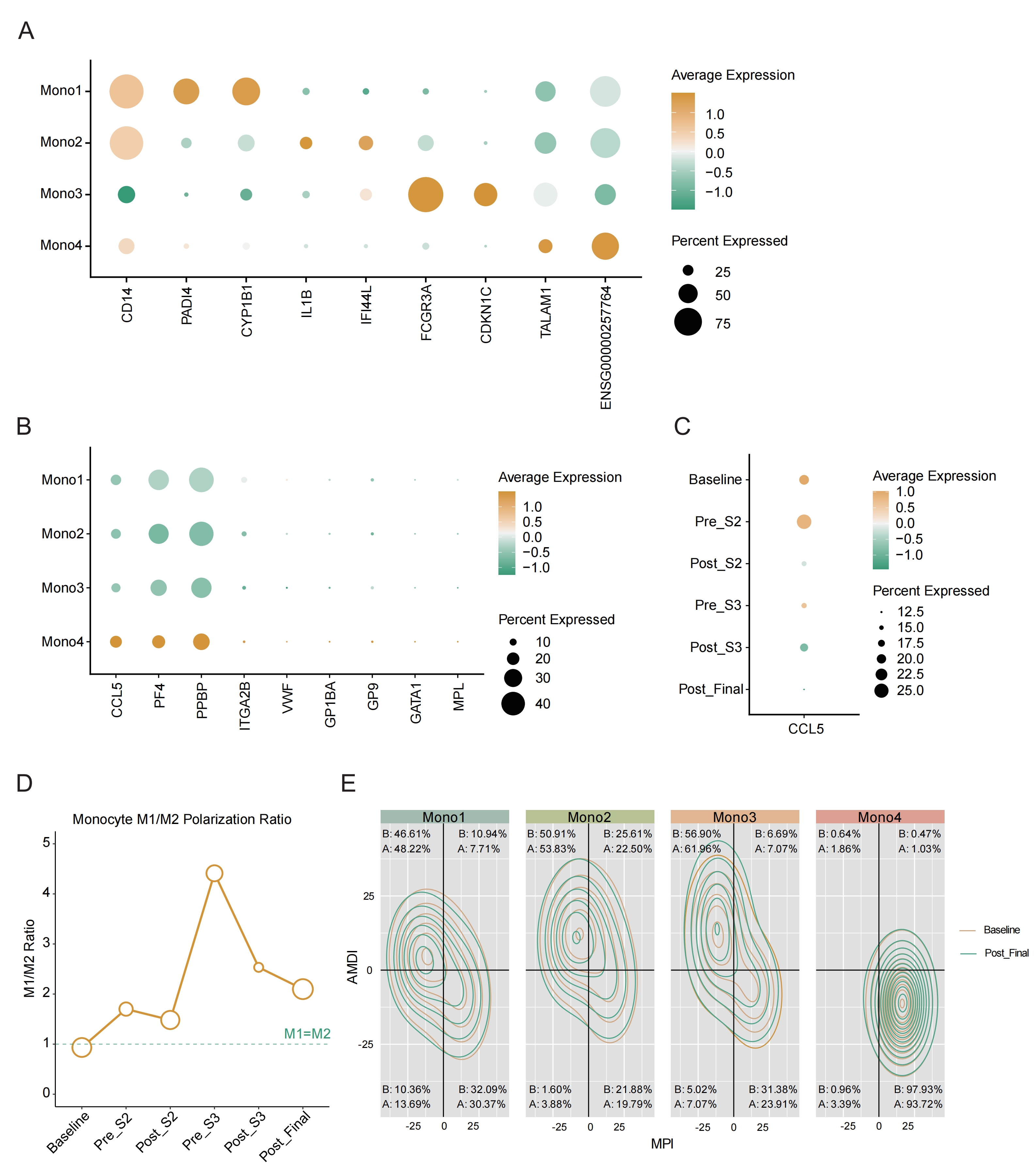

Supplement: Supplementary Figure 6 — Signatures, secretory phenotypes, and polarization dynamics of monocyte subsets during ALSS therapy. (A) Dot plot showing signature genes of monocyte subsets. (B) Dot plot showing secreted chemokines and platelet-associated genes in monocyte subpopulations. (C) Dot plot showing CCL5 expression across treatment time points. (D) Pseudobulk analysis of M1/M2 ratios in monocytes at different time points. Ratio was computed as (mean M1 module score)/(mean M2 module score) after per-timepoint averaging. Point size reflects cell number per timepoint. Dashed line denotes M1/M2 = 1. (E) Radar charts for each monocyte subset before (yellow lines) and after (green lines) comprehensive treatment, showing changes in the Monocyte Polarization Index (MPI) and the Differentiation Index (AMDI). Percentages denote the proportion of cells within each subset pre- (B) and post- (A) treatment. [file Image6.jpeg]

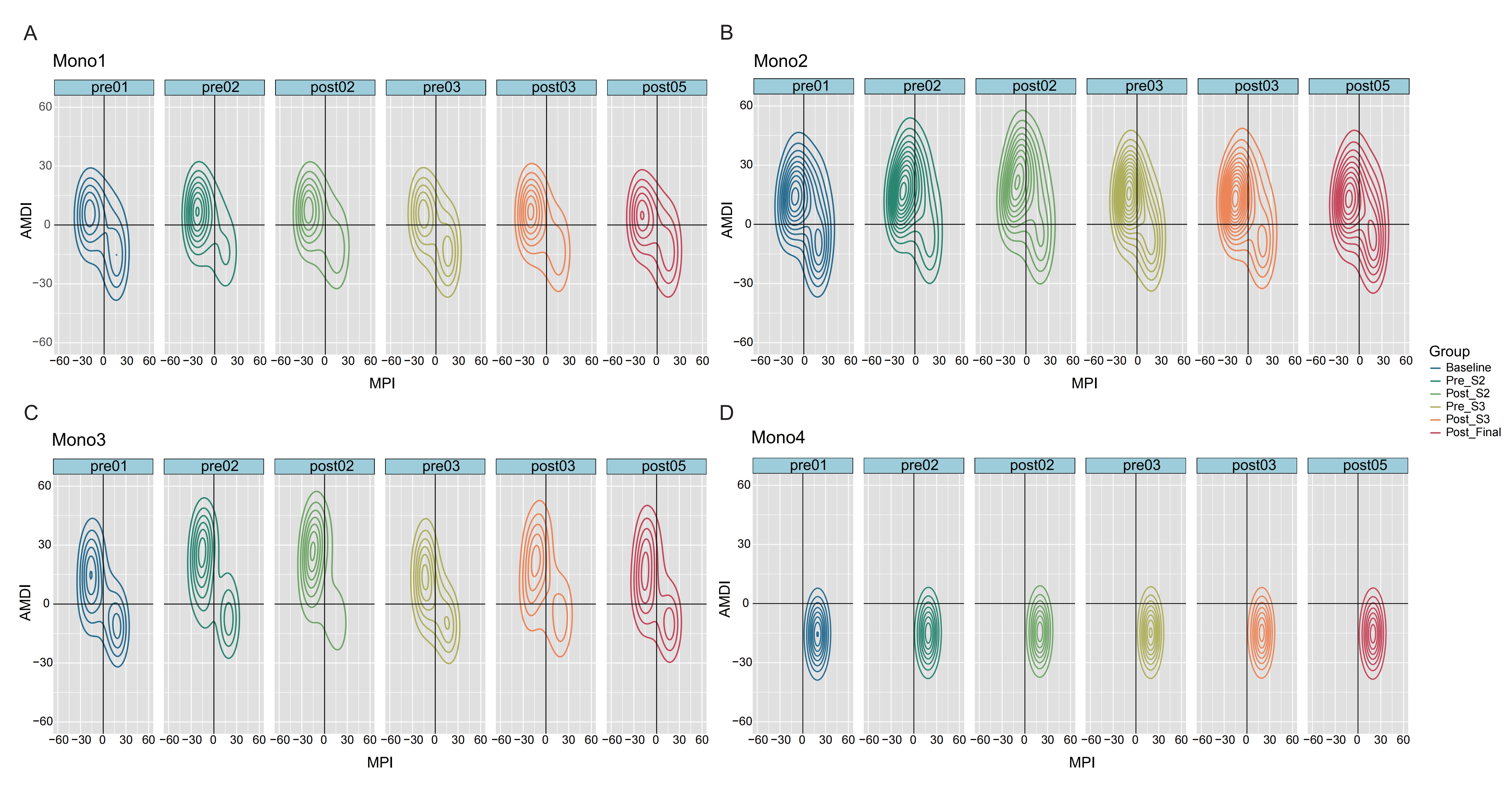

Supplement: Supplementary Figure 7 — Radar charts displaying the distribution of six time points for each monocyte subset (Mono1 to Mono4). [file Image7.jpeg]

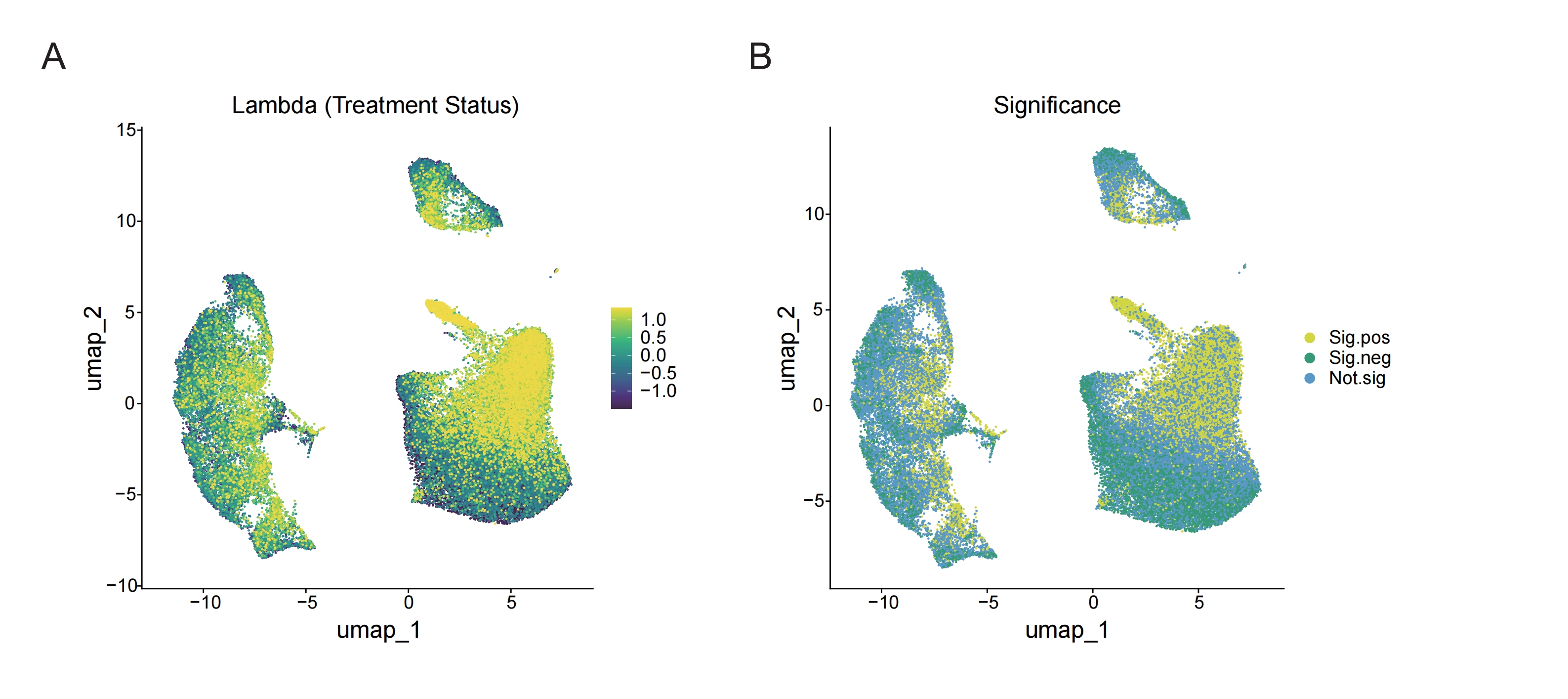

Supplement: Supplementary Figure 8 — SCIPAC integration of matched bulk and single-cell RNA-seq datasets from PBMCs. (A) per-cell lambda values. (B) Cells colored by significance. [file Image8.jpeg]
